# Dual use of novel tobacco products and socioeconomic paradox in smoking cessation: An age-period-cohort analysis of KNHANES data 2007–2022

**DOI:** 10.18332/tid/211616

**Published:** 2026-03-13

**Authors:** Hoang Le Tu, Xuan Dung Mai, Jin-kyoung Oh

**Affiliations:** 1 Department of Public Health and AI, National Cancer Center, Graduate School of Cancer Science and Policy, Goyang-si, Republic of South Korea; 2 Department of Biostatistics, Hanoi University of Public Health, Hanoi, Vietnam

**Keywords:** dual use, socioeconomic disparities, age-period-cohort analysis, smoking cessation intentions

## Abstract

**INTRODUCTION:**

Despite significant declines in smoking prevalence, South Korea faces stagnation linked to new tobacco product (NTP) adoption. This study examines sociodemographic and temporal drivers of smoking cessation intentions, with an emphasis on dual use of conventional cigarettes and NTPs.

**METHODS:**

We analyzed pooled secondary and nationally representative data from the Korea National Health and Nutrition Examination Survey 2007–2022 (n=37142) using an age period-cohort model via the Intrinsic Estimator. The analytic sample included adults aged ≥20 years who reported current smoking of conventional cigarettes or dual use with NTPs. Outcomes were intentions to quit within 1 and 6 months, adjusting for dual use status, sociodemographics, household factors, and applying survey weights.

**RESULTS:**

Dual users accounted for 6.4% of smokers, were more often college graduates (51.5% vs 37.0%, p<0.001) yet less likely to plan quitting. After adjusting for other covariates, cessation intentions peaked at the age of 35 years (adjusted prevalence odds ratio POR=1.111; 95% CI: 1.105–1.116) then declined by 23.1% by the age of 70 years. Following 2016 policy changes, intentions decreased overall (POR decreased from 1.012 to 0.994), while dual users showed an opposite period trend (POR increased from 0.966 to 1.022). Birth cohorts from 1947–1950 had higher intentions (POR=1.058; 95% CI: 1.051–1.064), contrasting with decreases in post-1955 cohorts (POR=0.969; 95% CI: 0.943–0.994).

**CONCLUSIONS:**

These findings demonstrate that socioeconomic and temporal factors are associated with quit intentions among Korean smokers. Future longitudinal and cross-country studies are needed to confirm these associations and to examine product-specific patterns and contextual influences, providing a broader understanding of how cessation intentions evolve in changing tobacco markets.

## INTRODUCTION

South Korea’s smoking prevalence has decreased sharply over thirty years, owing to the 1995 National Health Promotion Act, which introduced public-smoking bans, graphic warnings, and stricter rules on youth access^1^. From 1998 to 2022, men’s smoking rates fell from 66.3% to 30.0%, while women’s rates edged down from 6.5% to 5.0%^1^. However, progress stalled after 2017 with the rise of novel tobacco products (NTPs) – heated tobacco, electronic nicotine delivery systems, and other innovative products – now making up 14.8% of the market and still growing^2,3^. Overall smoking was 17.7% in 2022^1^, but quit intentions weakened markedly among dual users of cigarettes and NTPs, who account for 28.3% of smokers^4^. In December 2016, South Korea mandated graphic (pictorial) health warnings on all tobacco product packaging, requiring images and warning text to cover at least 50% of the front and back of cigarette packs^5^. Traditional cigarette sales fell by 1.8% per year after 2016, yet NTP sales increased 21.3% annually, due to marketing that presents them as modern and socially acceptable^3,6^. Smoking among women aged 20–39 years rose by 2.5% since 2015, in contrast to global trends, and military smokers remained high at 34.1% in 2022, highlighting persistent vulnerabilities^1^. Though early Korean tobacco laws were pioneering, current regulations are lagging: heated tobacco products have only a 90% tax compared to regular cigarettes and are exempt from outdoor bans, thus sustaining their appeal^7,8^.

Despite falling overall smoking rates, the impact of NTPs on quit behaviors remains unclear. Although marketed as safer, dual users of cigarettes and NTPs have 40% lower odds of intending to quit than those who smoke only cigarettes, and exclusive NTP users report the fewest quit attempts^9,10^. This pattern contradicts industry harm-reduction claims and reveals a socioeconomic paradox: well-educated, high-income individuals living in cities adopt heated tobacco and premium e-cigarettes most often but plan to quit less frequently^1,6^. Generational gaps add complexity with individuals born after 1990 using NTPs at higher rates (15.8% among those aged 30–39 years), while the elder cohorts stick with traditional cigarettes, reflecting past norms and policies^11^. Military personnel show persistent smoking linked to job stress and quit at rates 12 percentage points below civilians^1^. Rising smoking among women aged 20–39 years further underscores the need for gender-tailored interventions^1^.

Current research offers little detail on how NTPs influence quitting across life stages such as career changes or starting a family. Although age–period–cohort analyses show peak quit intentions at the age of 35 years, dual users experience smaller drops in motivation with age, implying NTPs disrupt normal quitting patterns^4,10^. Existing policies overlook the paradox in South Korea where higher education and income predict longer dual use instead of cessation – a trend not seen in Western countries^1,10^. This study uses age–period–cohort models to compare quit intentions across socioeconomic groups. It also evaluates how combining cigarettes with NTPs alters these trends by dampening age-related declines and reversing gains after 2016.

## METHODS

### Study design and data source

This is a pooled secondary dataset analysis using data from the Korea National Health and Nutrition Examination Survey (KNHANES), a nationally representative survey conducted annually from 2007 to 2022. KNHANES employs a multi-stage stratified probability sampling design to ensure representativeness of the population^12^. The analytic sample included adults aged ≥20 years who reported current smoking [conventional cigarettes or dual use (conventional cigarettes and NTPs users)], yielding an unweighted sample of 37142 participants.

### Variables and measurements

Primary outcomes were quit intentions categorized as no plan, plan within six months, or plan within one month, with a secondary outcome combining one- and six-month plans into a binary ‘intention to quit’ versus ‘no intention’ measure. The main exposure variable is concurrent use of conventional cigarettes and NTPs (heated tobacco or e-cigarettes). Covariates were categorized into temporal factors such as age (19–29, 30–39, 40–49, 50–59, 60–69, ≥70 years), six survey phases (2007–2009, 2010–2012 through 2022) and nine birth-cohort bands, and sociodemographics factors such as gender (male, female), income quintiles (1–lowest to 5–highest), education level (elementary school or lower, middle school, high school, college or higher), and type of household generation (1st or 2nd generation with different categories). All measures came from self-reported questionnaires under KNHANES protocols to ensure consistency^13^.

### Statistical analysis

*Age-period-cohort (APC) modeling*

The APC analysis was done using STATA BE version 19.5. The Intrinsic Estimator (IE) was applied to resolve collinearity among age, period, and cohort effects (by decomposing age, period, and cohort effects into independent components), as conventional constraints (e.g. equality constraints) introduce arbitrary biases. It was also recommended to avoid arbitrary assumptions, using orthogonal decomposition to isolate age, period, and cohort effects^14^. Survey-weighted multinomial logistic regression and logistic regression models were fitted using Stata 19.0’s *apc_ie* package^15^. The IE decomposes temporal effects into orthogonal components, ensuring unbiased estimates of age, period, and cohort contributions^14^. First, the survey-adjusted likelihood ratio test was used for univariable analysis. Then, we reported adjusted prevalence odds ratios (PORs) using multivariable logistic regression to assess associations between dual use and cessation intentions, adjusting for age, period, cohort, and sociodemographic factors. In this analysis, PORs were interpreted as the odds of having cessation intention compared with the those reported no cessation intention, consistent with cross-sectional study design recommendations^16^. All statistical tests were two-tailed, with a significance level set at α=0.05. For the ordinal logistic regression models, we also assessed the proportional odds assumption using the Brant test, and no violations were detected.

*Survey weighting*

All analyses incorporated KNHANES sampling weights, strata, and clusters to account for the complex survey design. Weighted percentages and odds ratios (ORs) with 95% confidence intervals (CIs) were computed using the *svy* suite in Stata 19.0, aligning with Centers for Disease Control and Preventions’ recommended practices for complex surveys^17^. Variance estimation was based on Taylor series linearization, the default method in Stata’s *svy* procedures, to ensure valid standard errors under the KNHANES’s design.

*Sensitivity analysis*

To assess robustness, we re-estimated the associations under several alternative modeling frameworks. First, for model robustness, we compared APC estimates across binary logistic regression (*logit* command), multinomial logistic regression (*mlogit* command), and models including interaction terms with survey phase (*glm* command). This allowed us to evaluate the consistency of temporal effect magnitudes and directions across different specifications. Second, for categorization sensitivity, we tested alternative groupings of temporal and demographic variables, including gender, 5-year age intervals, 3-year survey phases, and 10-year birth cohorts, to confirm the stability of APC patterns across classification strategies.

All sensitivity analyses retained the core APC methodology, complex survey weighting, and covariate adjustment protocols used in the primary models. Model fit was assessed using an F-adjusted mean residual goodness-of-fit test designed for complex survey data. In addition, residual deciles were derived by ranking covariate-adjusted residuals and dividing them into ten equal groups, which allowed assessment of whether model predictions systematically deviated from observed values.

### Ethical considerations

The KNHANES data collection procedures were reviewed and approved by the Institutional Review Board of the Korea Disease Control and Prevention Agency (KDCA). The ethical approval numbers varied by survey year: 2007-02CON-04-P (2007), 2008-04EXP-01-C (2008), 2009-01CON-03-2C (2009), 2010-02CON-21-C (2010), 2011-02CON-06-C (2011), 2012-01EXP-01-2C (2012), 2013-07CON-03-4C and 2013-12EXP-03-5C (2013), and 2013-12EXP-03-5C (2014). For the years 2015–2017, ethical approval was waived by the KDCA in accordance with Article 2 of the Bioethics and Safety Act, as KNHANES was classified as a legally mandated public health surveillance activity. Beginning in 2018, formal ethical oversight was reinstated. The KDCA Research Ethics Review Committee approved the 2018 survey under protocol 2018-01-03-P-A, with this approval extending to cover subsequent survey rounds through 2022. All participants provided written informed consent, and data were anonymized prior to public release.

## RESULTS

### Sociodemographic characteristics and prevalence of dual use among Korean smokers

Table 1 illustrates that the study population comprised 6.4% dual users (n=1839) among those currently smoking any types of cigarettes. Dual users were disproportionately younger, with 31.0% aged 19–29 years and 32.1% aged 39–39 years, compared to 15.7% and 19.8% of exclusive smokers, respectively (p<0.001). A socioeconomic contradictory pattern emerged: 51.5% of dual users held college degrees versus 37.0% of exclusive smokers, and 29.7% belonged to the highest income quintile versus 22.2% (p<0.001). Household structure further differentiated groups, with dual users more likely to reside in single-person households (15.1% vs 9.0%; p<0.001). Despite higher education and income, dual users reported lower concrete cessation intentions (69.5% with ‘no plan’ vs 66.3%; p=0.009).

**Table 1:** Characteristics of current smokers in KNHANES data, South Korea, 2007–2022 (N=37142)

*Characteristics*	*Conventional cigarettes* *users* *n (%)*	*Dual users (conventional* *cigarettes + NTPs)* *n (%)*	*Total* *n (%)*	p*
**Total** (weighted)	263368315 (93.6)	17896702 (6.4)	281265017 (100)	
**Total** (unweighted)	35303 (95.0)	1839 (5.0)	37142 (100)	
**Gender**				
Male	29612 (85.3)	1541 (86.0)	31153 (85.4)	0.492
Female	5691 (14.7)	298 (14.0)	5989 (14.6)	
**Age** (years)				
19–29	3626 (15.7)	493 (31.0)	4119 (16.7)	<0.001
30–39	5894 (19.8)	579 (32.1)	6473 (20.6)	
40–49	6579 (21.9)	465 (23.9)	7044 (22.1)	
50–59	6669 (20.2)	200 (9.9)	6869 (19.6)	
60–69	6510 (12.9)	82 (2.6)	6592 (12.3)	
≥70	6025 (9.4)	20 (0.5)	6045 (8.8)	
**Education level**				
Elementary school or lower	6408 (12.7)	33 (1.2)	6441 (12.0)	<0.001
Middle school	3985 (10.1)	87 (4.3)	4072 (9.7)	
High school	12403 (40.2)	754 (42.9)	13157 (40.4)	
College or higher	11366 (37.0)	908 (51.5)	12274 (37.9)	
**Household income** (quintiles)				
1 (Lowest)	6906 (15.3)	112 (5.9)	7018 (14.7)	<0.001
2	6902 (18.7)	233 (11.9)	7135 (18.3)	
3	7164 (21.8)	421 (22.7)	7585 (21.9)	
4	6933 (22.0)	520 (29.8)	7453 (22.5)	
5 (Highest)	7082 (22.2)	548 (29.7)	7630 (22.7)	
**Type of household**				
1st gen – Single	3502 (9.0)	285 (15.1)	3787 (9.4)	<0.001
1st gen – Couple	8849 (18.1)	216 (9.9)	9065 (17.6)	
1st gen – Other	574 (2.2)	70 (4.2)	644 (2.4)	
2nd gen – Couple + unmarried children	15070 (49.2)	907 (50.7)	15977 (49.3)	
2nd gen – Single parent + unmarried children	2111 (7.0)	175 (10.5)	2286 (7.3)	
2nd gen – Other	1894 (4.9)	65 (3.5)	1959 (4.8)	
3rd or more gen	3285 (9.5)	121 (6.2)	3406 (9.3)	
Don’t know/no response	13 (0.0)	0 (0.0)	13 (0.0)	
**Current HTPs use**	0 (0.0)	1042 (77.2)	1042 (13.2)	<0.001
**Current e-cigarettes use**	0 (0.0)	1054 (57.5)	1054 (5.8)	<0.001
**Type of smoking cessation intentions**				
Within 1 month	3234 (19.9)	267 (16.3)	3501 (19.5)	0.009
Within 6 months	2097 (13.8)	239 (14.2)	2336 (13.8)	
No concrete plan	10999 (66.3)	1203 (69.5)	12202 (66.7)	

KNHANES: Korea National Health and Nutrition Examination Survey. NTPs: novel tobacco products (heated tobacco, electronic nicotine delivery systems, and other innovative products).

* Survey-adjusted likelihood ratios test.

### Age, period, and cohort effects on overall smoking cessation intentions

**Figure 1:**
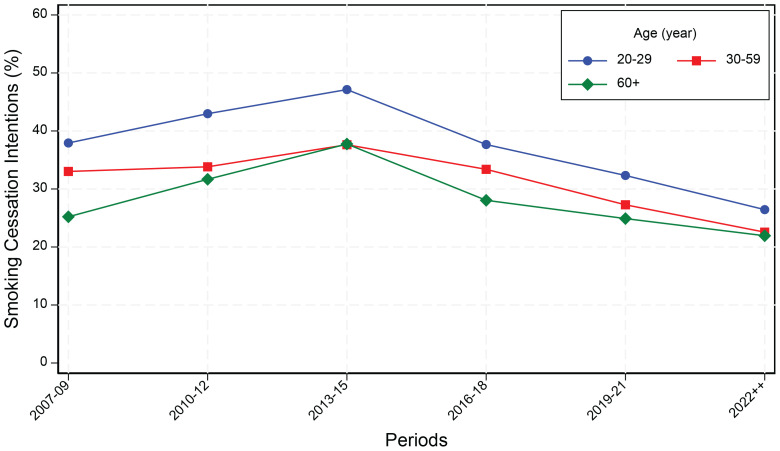
Smoking cessation intentions by age and period among current smokers in Korea National Health and Nutrition Examination Survey (KNHANES) data, South Korea, 2007–2022 (N=37142)

Figure 1 illustrates the trends in smoking cessation intentions across three age groups (20–29, 30–59, and ≥60 years) in six periods from 2007–2009 to 2022 and later. The figure reveals a consistent pattern where younger adults (aged 20–29 years) maintained higher cessation intentions throughout all periods compared to middle-aged (aged 30–59 years) and the elders (aged ≥60 years), with the highest intentions (47%) observed in 2013–2015. All age groups demonstrated a similar trajectory, with cessation intentions increasing from 2007–2009 to peak in 2013–2015, followed by a steady decline through 2022 and later, where intentions reached their lowest levels (22–26%) across all age groups.

**Figure 2:**
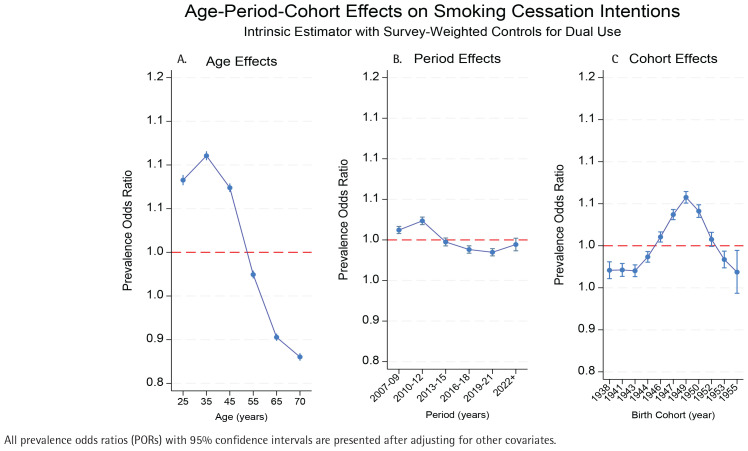
Age-period-cohort effects on smoking cessation intentions of dual users among current smokers in Korea National Health and Nutrition Examination Survey (KNHANES) data, South Korea, 2007–2022

In Figures 2A-2C, cessation intentions form an inverted U-shape, peaking at the age of 35 years (POR=1.111; 95% CI: 1.105–1.116) and declining thereafter, falling below unity after the age of 50 years and reaching 0.880 (95% CI: 0.876–0.885) by the age of 70 years (a 23.1% drop from the peak) all highly significant (p<0.001). Temporal influences were modest: intentions were highest in 2010–2012 (POR=1.023; 95% CI: 1.019–1.028) and slightly positive in 2007–2009 (POR=1.012; 95% CI: 1.008–1.017), but declined significantly in 2016–2018 (POR=0.988; 95% CI: 0.984–0.993) and 2019–2021 (POR=0.985; 95% CI: 0.980–0.989). Effects in 2013–2015 and after 2022 were not significant. Cessation intentions were lowest among 1938–1944 cohorts, peaked for those born in 1949 (POR=1.058; 95% CI: 1.051–1.064), then declined in later cohorts. Cohorts 1947–1950 maintained PORs above 1.035, whereas the 1955 cohort fell to 0.969 (95% CI: 0.943–0.994). Cohort variation (9.1%) exceeded period effects (3.8%) but was smaller than age effects (23.1%). Detailed results are presented in Supplementary file Table S2.

### Temporal determinants of immediate and intermediate smoking cessation intentions

**Figure 3:**
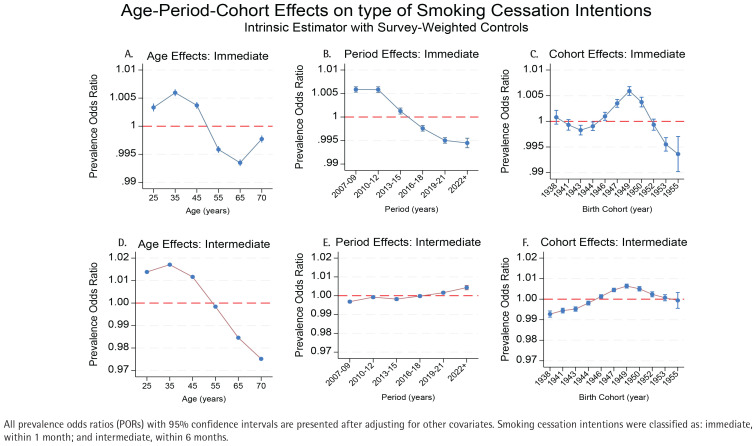
Age-period-cohort effects on smoking cessation intentions by type of smoking cessation intentions among current smokers in Korea National Health and Nutrition Examination Survey (KNHANES) data, South Korea, 2007–2022

Figures 3A-3F presents the age-period-cohort effects on two distinct cessation intention timeframes: immediate (within 1 month) and intermediate (within 6 months). Immediate quit intentions follow a gentle inverted U-shape, peaking at the age of 35 years (POR=1.006; 95% CI: 1.005–1.007) and varying only 1.3% across ages. In contrast, intermediate intentions rise to a POR=1.017 (95% CI: 1.016–1.018) at the age of 35 years then fall to 0.975 (95% CI: 0.974–0.976) by the age of 70 years, a 4.2% drop. Immediate intentions were strongest in 2007–2012 (POR=1.006 both phases, 95% CI: 1.005–1.007) before declining to 0.995 (95% CI: 0.994–0.996) in 2019–2021, and 0.994 (95% CI: 0.993–0.995) in 2022 and later. Intermediate intentions show the reverse: low odds early (POR=0.997; 95% CI: 0.996–0.998 in 2007–2009) and rising to 1.002 (95% CI: 1.001–1.003) in 2019–2021 and 1.004 (95% CI: 1.003–1.005) after 2022. For immediate intentions, effects are flat for 1938–1944 cohorts, peak at 1949 (POR=1.006; 95% CI: 1.005–1.007), then down to 0.994 (95% CI: 0.993–0.996) by 1955. Intermediate intentions mirror this but with stronger gains in 1947–1952 cohorts. Both measures climax in the 1949–1950 cohorts, while the 1955 cohort shows a substantial drop only for immediate plans. The confidence intervals shown in Supplementary file Table S3 indicate greater precision in estimating age effects compared to cohort effects, particularly for recent cohorts.

### Dual use as a modifier of temporal patterns in smoking cessation intentions

**Figure 4:**
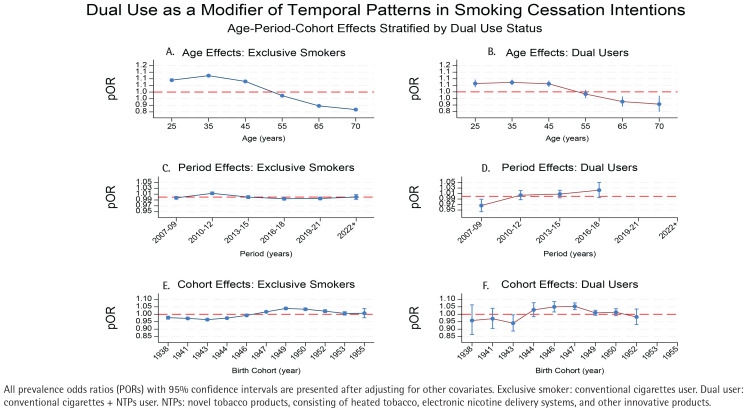
Age-period-cohort effects on smoking cessation intentions by type of smoker among Korean current smokers in Korea National Health and Nutrition Examination Survey (KNHANES) data, South Korea, 2007–2022

Figures 4A-4F presents the stratified APC effects by tobacco use status, revealing substantial differences in temporal patterns between exclusive smokers and dual users. For age effects, exclusive smokers demonstrated a pronounced inverted U-shaped pattern, with cessation intentions peaking at the age of 35 years (POR=1.124; 95% CI: 1.119–1.129) before declining steadily to a minimum at the age of 70 years (POR=0.868; 95% CI: 0.863–0.872). This represents a 25.6% reduction in cessation odds across the life course. In contrast, dual users exhibited a flatter age trajectory through middle age (25–45 years), maintaining relatively consistent prevalence odds ratios (POR range: 1.061–1.072), with a more modest decline at the age of 70 years (POR=0.907; 95% CI: 0.848–0.970). In particular, the age-related decline in cessation intentions was significantly attenuated among dual users compared to exclusive smokers (15.4% vs 25.6% reduction from peak to minimum).

The quit intentions of exclusive smokers peaked in 2010–2012 (POR=1.013; 95% CI: 1.008–1.017) before declining in 2016–2018 (POR= 0.994; 95% CI: 0.990–0.999) and 2019–2021 (POR=0.995; 95% CI: 0.991–1.000). Dual users showed the reverse: low intentions early (2014) (POR =0.966; 95% CI: 0.944–0.989) but rising to 1.022 (95% CI: 0.995–1.050) by 2022. Among exclusive smokers, early cohorts (1938–1946) had reduced intentions (lowest at 1943, POR=0.964; 95% CI: 0.957–0.971), while cohorts born 1947–1952 peaked at 1949 (POR=1.039; 95% CI: 1.032–1.046). The cohort trends of dual users were more variable, with wider CIs in the elder cohorts. In particular, the 1949 cohort was positive for exclusive smokers but negative for dual users (POR=0.940; 95% CI: 0.887–0.997). Recent cohorts (1952–1955) showed increased intentions for dual users (POR=1.053; 95% CI: 1.031–1.076) but flat or lower odds for exclusive smokers. Gender was also considered as a modifier of temporal patterns in smoking cessation intentions (Supplementary file Figure S1). Similar patterns between genders were observed by different age groups, birth cohorts and periods.

### Model comparison statistics

We conducted a residual decile analysis using covariate-adjusted residuals to examine whether systematic patterns in the residuals were associated with cessation intentions (Supplementary file Table S1). The analysis showed significant associations between residual deciles and cessation intentions in the multinomial regression (all p<0.001), suggesting that residual patterns may reflect unmeasured determinants of quit timing. In contrast, binary and interaction models showed no such effects, indicating that covariate adjustment was adequate for predicting overall cessation intent, though additional modeling may be needed to better capture specific aspects of quit timing.

## DISCUSSION

### Key findings

This study identified three main findings regarding smoking cessation intentions among Korean adults. First, a sociodemographic contradictory pattern emerged: dual users of conventional cigarettes and NTPs exhibited higher socioeconomic status (SES) – including a higher level of education and higher income level – yet demonstrated significantly lower concrete cessation intentions, these results are in agreement with some studies^4,18^. But this contrasts with global patterns where higher SES typically is associated with cessation success, highlighting the unique influence of targeted NTP marketing to affluent demographics in Korea^19,20^. Second, APC analysis revealed distinct temporal determinants of cessation intentions. Age effects peaked at the age 35 years (POR=1.111; 95% CI: 1.105–1.116), declining through the age of 70 years, reflecting life-course transitions and entrenched addiction patterns in the elders^21,22^. Period effects showed significant declines post-2016 (POR decreased from 1.012 to 0.994), coinciding with reduced policy innovation and NTP proliferation, while cohort effects highlighted generational shifts, with peak intentions among 1947–1950 birth cohorts (POR=1.058; 95% CI: 1.051–1.064) and declines in post-1955 cohorts (POR=0.969; 95% CI: 0.943–0.994), which are consistent with previous results^21,22^. These patterns also align with a Japanese study documenting similar cohort vulnerabilities to tobacco marketing^7^.

Lastly, dual use significantly modified temporal patterns. Dual users exhibited attenuated age gradients and reversed period trends (post-2016, POR=1.022; 95% CI: 0.995–1.050 vs 0.995; 95% CI: 0.991–1.000 for exclusive smokers), suggesting industry success in framing NTPs as harm-reduction tools despite evidence of prolonged nicotine dependence^4,19^. This contrasts with European studies showing uniform APC patterns across user types, likely due to stricter regulations on novel products like NTPs^7,19^. The findings reflect a dual-use contradictory pattern amplified by NTP marketing. While dual use itself (concurrent conventional + NTP use) sustains nicotine dependence, the core driver is NTPs’ socioeconomic stratification. Higher SES groups disproportionately adopt NTPs due to targeted narratives framing these products as ‘safer’ or ‘modern’ alternatives^23^. For example, 41.4% of college-educated dual users adopted NTPs to ‘reduce conventional cigarette use’, yet longitudinal data show minimal cessation success. This aligns with global trends where NTPs normalize tobacco use among affluent populations, particularly in Asia^24^.

### Dual use contradictory pattern: socioeconomic advantage versus cessation resistance

The paradoxical association between higher SES and reduced cessation intentions among dual users in Korea reflects a nuanced interplay between dual-use behavior and NTP dynamics, with the core driver being an NTP contradictory pattern amplified by dual use. Globally, lower SES groups face structural barriers to cessation, such as financial strain and limited healthcare access^25-27^, yet Korean dual users – characterized by higher education and income – exhibited lower quit intentions. This contradiction aligns with Japan’s NTP landscape, where heated tobacco products (HTPs) adoption is concentrated among educated city dwellers, but contrasts with Europe’s uniform smoking declines across SES groups under stringent NTP regulations^28^. Three factors explain this contradictory pattern. First, targeted NTP marketing to high-SES groups exploits health literacy gaps among high-SES groups by framing these products as ‘reduced-risk’ alternatives. In Korea, 41.4% of college-educated dual users adopted NTPs explicitly to ‘reduce conventional cigarette use’, yet longitudinal data show minimal cessation success^29^. This reflects industry success in positioning NTPs as sustainable substitutes rather than cessation tools, exploiting health literacy gaps among high-SES populations^30^. Second, while NTPs drive the contradictory pattern, dual use exacerbates it by reinforcing nicotine dependence. Affluent social environments further normalize this behavior: the partnerships of Korea Tobacco & Ginseng (KT&G, also branded as Korea Tomorrow & Global) with transnational firms (e.g. with Philip Morris International) and premium NTP designs (e.g. AI-enabled devices) frame NTPs as symbols of technological sophistication, embedding them into high-SES lifestyles^29,30^. Third, misplaced harm reduction beliefs persist despite evidence of comparable nicotine dependence and cardiovascular risks between NTPs and conventional cigarettes^7^. This contrasts sharply with LMICs, where lower SES is associated with cessation failure due to financial strain, limited healthcare access, and pro-smoking social contexts^25,26^. In Korea, however, the tobacco industry has inverted this dynamic by capitalizing on socioeconomic privilege. While lower SES smokers globally face structural barriers (e.g. stress-induced cravings, lack of cessation support)^26,27^, high-SES dual users in Korea are ensnared by sophisticated marketing narratives that equate HTPs use with health-conscious decision-making^28,30^.

Dual use as an effect modifier: Divergent temporal patterns in East Asia versus uniform trends in Europe

The modifying effect of dual tobacco use on APC patterns observed in Korea contrasts sharply with European studies reporting uniform temporal trends across user types. While Korean dual users exhibited attenuated age gradients and reversed period effects post-2016, European analyses, such as those from the Ageing Lungs in European Cohorts consortium, found no significant APC differences between dual users and exclusive smokers^31^. For instance, smoking initiation rates during late adolescence (aged 16–20 years) declined uniformly across all user groups in Europe, irrespective of product type, with no evidence of diverging cohort effects among dual users^31^. This discrepancy stems from stark regulatory and market differences. In Europe, the 2016 Tobacco Products Directive (TPD) imposed uniform restrictions on conventional and novel products, including flavor bans, advertising prohibitions, and standardized health warnings, mitigating differential temporal effects^32^. By contrast, Korea’s delayed regulation of HTPs allowed industry marketing to exploit socioeconomic disparities, targeting affluent, educated demographics with ‘harm reduction’ narratives^33^. European dual users, while prevalent (68% of HTP users are dual consumers), show no significant APC deviations, as policies like the TPD reduced product-specific behavioral segmentation^34^. The uniform European patterns may also reflect transitional dual use, where e-cigarettes serve as short-term cessation aids rather than sustained alternatives. In Europe, only 1.8% of dual users maintained long-term dual consumption, compared to 69.5% in Korea^34^. Korea’s post-2016 resurgence in dual users’ cessation intentions parallels industry efforts to rebrand HTPs as cessation tools – a strategy less effective in Europe due to pre-emptive bans on health claims^32^.

### Strengths and limitations

This study uses nationally representative KNHANES survey weights, ensuring its findings apply to Korean adults who smoke. Employing the Intrinsic Estimator for age-period-cohort analysis overcomes collinearity issues common to standard regression, yielding more reliable temporal estimates. Additionally, distinguishing immediate from intermediate quit intentions contributes to understanding of how time horizons differently affect short- and long-term planning – an issue that has received limited attention in tobacco control research.

The study has some limitations. First, the cross-sectional design prevents causal inference, and the possibility of reverse causation cannot be excluded (e.g. smokers with lower cessation intention may be more likely to adopt novel tobacco products rather than dual use influencing quit intentions). Second, residual confounding remains possible, as unmeasured variables such as psychological distress or exposure to comprehensive tobacco marketing may partly explain the observed associations. Third, although the analysis used complex survey weights, the findings are specific to the Korean context and may have limited generalizability to other countries with different tobacco markets and regulatory environments. Fourth, grouping all NTPs into one category may obscure product-specific effects, such as differences between heated tobacco and e-cigarettes. Fifth, reliance on selfreported intentions could introduce social desirability bias, especially among educated dual users, despite moderate validation against actual quit attempts^35^.

These limitations highlight opportunities for future research, including longitudinal cohorts that track actual cessation outcomes, product-specific analyses and cross-country comparisons to better capture global diversity in tobacco use patterns. Despite these constraints, the study’s integration of socioeconomic, temporal, and behavioral dimensions provides a replicable framework for analyzing complex determinants of smoking cessation in rapidly evolving nicotine markets.

## CONCLUSIONS

This age-period-cohort analysis of Korean dual users demonstrates the contradiction that higher socioeconomic status is associated with weaker quit intentions. Despite greater resources, cessation intentions among dual users remained remarkably stable through middle age before rebounding after 2016. These findings suggest that socioeconomic and temporal factors play an important role in shaping cessation intentions. Further longitudinal and cross-country studies are needed to confirm these associations and to disentangle product-specific differences between heated tobacco products and e-cigarettes. Future research may also explore how marketing exposure, policy environments, and psychosocial factors interact with socioeconomic status to shape cessation intentions, providing a more comprehensive understanding of these complex dynamics.

## Supplementary Material



## Data Availability

The data supporting this research are available from the following source: KDCA website: https://knhanes.kdca.go.kr/knhanes/rawDataDwnld/rawDataDwnld.do
