# Supplementary material for: Dual use of novel tobacco products and socioeconomic paradox in smoking cessation: An age-period-cohort analysis of KNHANES data 2007–2022
Source: Tob Induc Dis. 2026 Mar 13;24:10.18332/tid/211616. doi: 10.18332/tid/211616 (PMC13005606; doi:10.18332/tid/211616)
Supplement: Supplementary file 1 [file TID-24-36-s1.pdf]

Dual Use of Novel Tobacco Products and Socioeconomic Paradox in Smoking Cessation: An Age-Period-Cohort Analysis of KNHANES Data (2007–2022)

Table S1. Associations between residual deciles and smoking cessation intentions across modeling approaches

	Binary Model	Multinomial Model	Interaction Model
Residual Decile 1	-0.00120	2.396***	0.00687
(Smallest residuals - best model fit)	(-0.09)	(147.02)	(0.65)
Residual Decile 2	0.00218	2.327***	-0.0145
	(0.19)	(113.89)	(-1.17)
Residual Decile 3	0.0139	2.275***	-0.00862
	(0.93)	(115.78)	(-0.66)
Residual Decile 4	0.00396	2.226***	0.0253
	(0.26)	(111.39)	(1.63)
Residual Decile 5	-0.00996	2.211***	0.0105
	(-0.94)	(108.43)	(0.84)
Residual Decile 6	-0.00368	2.184***	-0.0195
	(-0.37)	(89.53)	(-1.47)
Residual Decile 7	0.00740	2.177***	-0.00447
	(0.68)	(106.63)	(-0.39)
Residual Decile 8	-0.0263	2.118***	0.0159
	(-1.50)	(68.65)	(1.22)
Residual Decile 9	0.0116	2.078***	-0.00977
	(0.51)	(55.21)	(-0.63)
Residual Decile 10	0.0469	1.903***	0.00102
(Largest residuals - poorest model fit)	(1.40)	(29.65)	(0.04)
Sample size	17306	17306	17306
F-statistic	0.6368	10025.819	0.9601
p-value	0.7663	<0.001	0.4712

Residual deciles were derived by ranking residuals from the APC model and dividing them into ten equal groups, which allowed assessment of whether model predictions systematically differed from observed values. t statistics in parentheses. Goodness-of-fit p-values from F-adjusted test for survey data.

* $p < 0.05$, ** $p < 0.01$, *** $p < 0.001$

Table S2. Age-Period-Cohort effects on smoking cessation intentions

	Adjusted Odds Ratio	Standard error	p-value	95% lower bound	95% upper bound
Age: 20-29	1.083	0.003	<0.001	1.077	1.089
Age: 30-39	1.111	0.003	<0.001	1.105	1.116
Age: 40-49	1.074	0.003	<0.001	1.069	1.079
Age: 50-59	0.975	0.002	<0.001	0.970	0.979
Age: 60-69	0.903	0.002	<0.001	0.899	0.907
Age: 70++	0.880	0.002	<0.001	0.876	0.885
Period: 2007-09	1.012	0.002	<0.001	1.008	1.017
Period: 2010-12	1.023	0.002	<0.001	1.019	1.028
Period: 2013-15	0.998	0.002	0.315	0.993	1.002
Period: 2016-18	0.988	0.002	<0.001	0.984	0.993
Period: 2019-21	0.985	0.002	<0.001	0.980	0.989
Period: 2022++	0.994	0.004	0.153	0.987	1.002
Cohort: 1938	0.971	0.005	<0.001	0.961	0.981
Cohort: 1941	0.971	0.004	<0.001	0.964	0.979
Cohort: 1943	0.970	0.004	<0.001	0.963	0.977
Cohort: 1944	0.987	0.003	<0.001	0.980	0.993
Cohort: 1946	1.010	0.003	0.001	1.004	1.016
Cohort: 1947	1.037	0.003	<0.001	1.031	1.043
Cohort: 1949	1.058	0.004	<0.001	1.051	1.064
Cohort: 1950	1.041	0.004	<0.001	1.034	1.049
Cohort: 1952	1.008	0.004	0.076	0.999	1.016
Cohort: 1953	0.984	0.005	0.001	0.974	0.994
Cohort: 1955	0.969	0.013	0.018	0.943	0.994
Intercept	0.485	0.001	<0.001	0.483	0.487

All prevalence Odds Ratios (pOR) are presented after adjusted for other covariates with 95% confidence intervals.

Table S3. Age-Period-Cohort effects on smoking cessation intentions by type of smoking cessation intentions

	Immediate			Intermediate		
	**Adjusted Odds Ratio**	**95% lower bound**	**95% upper bound**	**Adjusted Odds Ratio**	**95% lower bound**	**95% upper bound**
Age: 20-29	1.003	1.003	1.004	1.014	1.013	1.015
Age: 30-39	1.006	1.005	1.007	1.017	1.016	1.018
Age: 40-49	1.004	1.003	1.004	1.012	1.011	1.012
Age: 50-59	0.996	0.995	0.996	0.998	0.998	0.999
Age: 60-69	0.993	0.993	0.994	0.985	0.984	0.985
Age: 70++	0.998	0.997	0.998	0.975	0.974	0.976
Period: 2007-09	1.006	1.005	1.006	0.997	0.996	0.997
Period: 2010-12	1.006	1.005	1.006	0.999	0.999	1.000
Period: 2013-15	1.001	1.001	1.002	0.998	0.998	0.999
Period: 2016-18	0.998	0.997	0.998	1.000	0.999	1.000
Period: 2019-21	0.995	0.994	0.996	1.002	1.001	1.002
Period: 2022++	0.994	0.993	0.995	1.004	1.003	1.005
Cohort: 1938	1.001	0.999	1.002	0.993	0.991	0.994
Cohort: 1941	0.999	0.998	1.000	0.994	0.993	0.996
Cohort: 1943	0.998	0.997	0.999	0.995	0.994	0.996
Cohort: 1944	0.999	0.998	1.000	0.998	0.997	0.999
Cohort: 1946	1.001	1.000	1.002	1.001	1.000	1.002
Cohort: 1947	1.003	1.003	1.004	1.004	1.004	1.005
Cohort: 1949	1.006	1.005	1.007	1.006	1.005	1.007
Cohort: 1950	1.004	1.003	1.005	1.005	1.004	1.006
Cohort: 1952	0.999	0.998	1.000	1.002	1.001	1.004
Cohort: 1953	0.996	0.994	0.997	1.001	0.999	1.002
Cohort: 1955	0.994	0.990	0.997	0.999	0.996	1.003
Intercept	1.216	1.216	1.217	1.142	1.141	1.142

All prevalence Odds Ratios (pOR) are presented after adjusted for other covariates with 95% confidence intervals. Smoking cessation intentions were classified as: Immediate: within 1 month – Intermediate: within 6 months

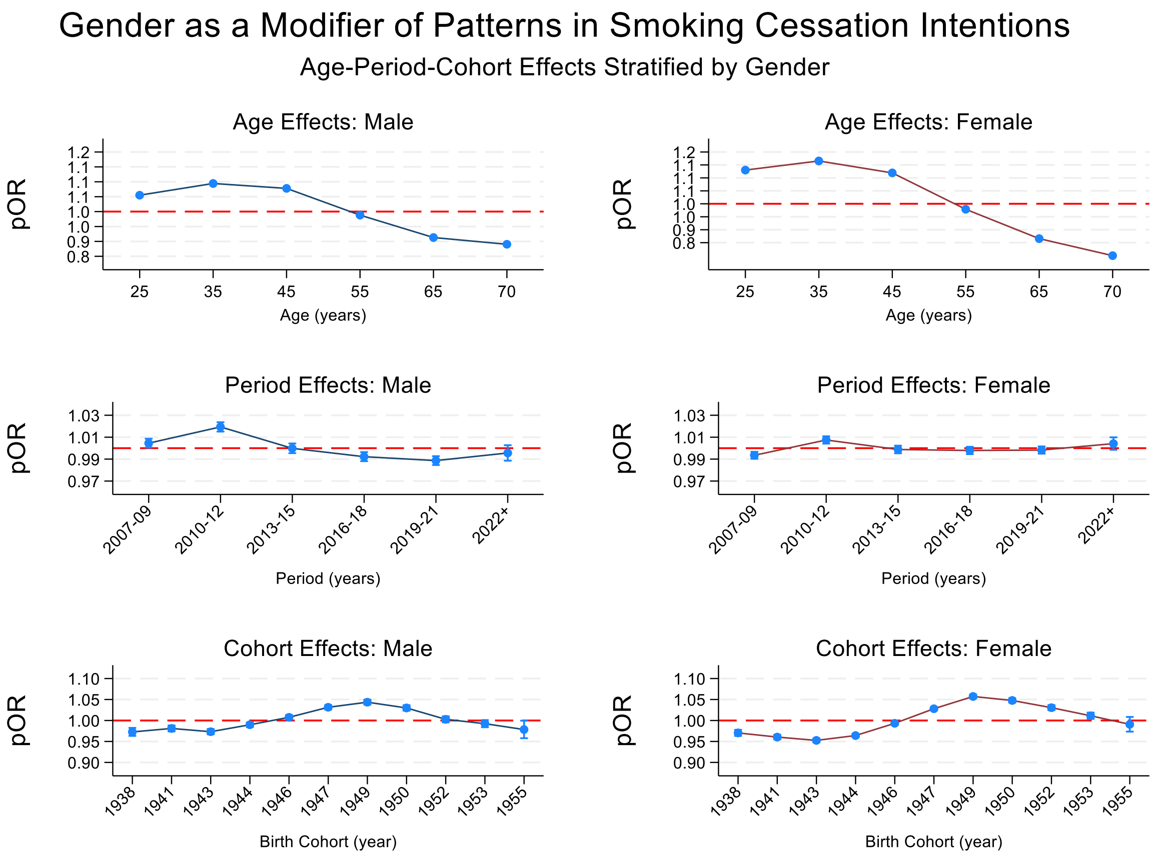

All prevalence Odds Ratios (pOR) are presented after adjusted for other covariates with 95% confidence intervals.

Figure S1. Age-Period-Cohort effects on smoking cessation intentions by gender

© 2026 Le Tu H. et al.
